# Hospital Progress and Finance

**Published:** 1924-11

**Authors:** 


### HOSPITAL PROGRESS AND FINANCE.

### A £10,000 GRANT AT LEEDS.

The Leeds City Council has unanimously agreed to make a grant of £10,000 to the General Infirmary and the Leeds Public Dispensary in consideration of an agreement between the Council and the committee of each. The hospital and dispensary are to receive sick persons within the city requiring treatment, and the grant will be distributed in the proportions that the Finance and Parliamentary Committee of the Council may decide. The Leeds Maternity Hospital is not included because it is not covered by the Public Health Act of 1875, under which the grant is made. Everyone welcomes this grant in return for services to be rendered. It is made also at a time when the Lord Mayor is trying to reduce the debt of the hospitals, and when, too, the waiting list at the General Infirmary runs, we believe, into four figures. The grant will help the debt, and thus indirectly make extension possible later. Whether the grant will become annual or not is yet undetermined, and Sir Charles Wilson, who moved the resolution, said that the law restricts the Corporation, and referred to the promotion of a Bill to amend it. Already, we understand, nearly threequarters of the employers in the city contribute to their hospital fund, and if all come in it is believed that an annual grant from the Corporation may not be needed. Though the grant was not opposed, the word "municipalisation" was mentioned by some speakers. We cannot too often remind them that this polysyllable means a subsidy, while a contribution in return for definite services is a different thing. The difference is vital to the voluntary system, and it is a pity that people should confuse themselves over a distinction obvious enough.

### ANOTHER QUESTION FOR LEEDS.

The supplementary scheme, not long ago added in Leeds to the plan by which employers contribute to the hospitals 2s. for each member of their staffs, seems to be making headway. It is one of those endeavours aimed at that section of the community, so elusive to the hospital secretary, which is included in the different ranks of professional men, business people, retailers and the like. It tries to reach heads of departments rather than their staffs, for whom the oldest of these funds, after all, provides a not unfitting organisation. Once more the principle of sub-division is invoked, and this divides Leeds into twenty sections, containing four thousand firms. Each section is referred to its own sub-committee, which has the delicate task of assessing each firm for the amount of its desired and reasonable contribution. Mr. J. F. Braime, the veteran organiser of these efforts in Leeds, once more has taken up his pen. In a statement that he has issued in support of the supplementary scheme, he employs the business argument which, in effect, says: either organise voluntary effort on behalf of our hospitals or expect to be taxed and rated for the State or municipal institutions that will replace them if they are allowed to fail. With admirable directness Mr. Braime makes the position of his own firm into a test case. He explains how, at present, its contribution is £50 a year, and that 2s. in the pound upon its rateable value would amount to £325, or six and a-half times its voluntary contribution. A similar argument is applied to the professional men and business people, a hundred of whom received, and apparently accepted, a proposal to contribute when Mr. Braime laid the plan before them. Will the rest follow suit?

### COTTAGE HOSPITALS AND LOCAL COMMITTEES.

At the end of the third year of its existence the Surrey and Croydon Voluntary Hospitals Committee, of which Lord Cave is chairman, has some interesting facts to report on cottage hospitals and other topics. The committee has found cottage hospitals to be indispensable, and that they are better equipped than they were ten years ago. Therefore, runs the argument, beware of too much centralisation. There appears small risk of this in Surrey, where, we learn, every hospital in the county is now paying its way. To this admirable result the committee has no doubt contributed, for it explains their solvency by the supervision that is now possible, whereby expenses have been reduced and income maintained or enlarged. The committee also reports that there is little overlapping in collections, and that each locality is eager to maintain its own hospital independently. Thus combined appeals are found unsuited to this excellent parochial spirit, in the country parts of the county at least. Here, as elsewhere, co-operative purchase of supplies is not favoured, because the practice is as complicated as the theory sounds simple. These results have been cheaply gained at a cost of £30, which was the total of the committee's expenses in the year now over.

### "SAVED FROM CERTIFICATION."

The value of the out-patient clinic now becoming attached to many mental hospitals is apparent in the first report on this work at the Dorset Mental Hospital. Dr. Peachell, the medical superintendent, states that the Saturday morning sessions, begun on October 14, 1922, and continued (as they still are) to December 31, 1923, showed 246 attendances by fifty-two new patients. This quiet beginning was better than Dr. Peachell had hoped, and he states, as we can believe, the results to be more important than can be expressed, under bald categories of "relieved" or "not improved," on paper. Even advanced cases, such as imbeciles, received advice of benefit to their future and of value to their relations. "In at least six cases," we are told, "treatment at the clinic saved the patient from certification." Now that the clinic is better known, more cases, and more cases suitable for treatment, are being received, and the moral that Dr. Peachell draws, " one must be willing to treat any patient sent," even the least hopeful, is one that should not be lost upon the general public. Indeed, the record of these clinics, of which that of Dorset is doubtless typical, can hardly be made known too widely in the interests of prevention and the forestallment of relapse.

### A REMARKABLE BENEFACTOR.

Though bequests to hospitals are not the chief items in the big sum of £112,000 that the late Mr. James Parr, of Higher Openshaw, Manchester, left to charities, his will deserves to be recorded, for he was a remarkable man. Beginning as an office boy in a firm of bleachers at Manchester, he became its head, retired from business twenty-five years ago, and has lately died at the age of 87, leaving a fortune of £197,000. This has much impressed lay journalists, and so their readers, because at the time of his death Mr. Parr, a bachelor, was living in a six-roomed house letting at 13s. a week. It has apparently seemed odd to the average, and therefore poor, man that Mr. Parr did not spend to the limit of his means and inhabit what the house agents call a princely mansion. We cannot share this surprise. Surely it would have been much stranger had Mr. Parr sacrificed his personal preferences merely to copy the expenditure of other rich men. Mr. Parr had no family responsibilities, and he lived as he liked. Many charities, including some hospitals, benefit considerably by his will: St. Mary's Hospital receives £6,000 now, and a large sum eventually, and Ancoats Hospital £6,000 also.

### THE MISERABLE TICKET SYSTEM.

Old anomalies, like old abuses, die with difficulty, and the miserable ticket system is succumbing only slowly to continuous exposure of its defects. It seems, like injustice, to curse both him who buys and him who seeks a ticket. At Birmingham an annual donor of two guineas is unable, it seems, to obtain a ticket because he cannot promise to continue this annual donation for an indefinite number of years. The unfortunate man was told, he says, that if donors received tickets, subscribers would diminish and possibly find, through the greater supply, some tickets left on their hands. This argument is abominable. The voluntary hospitals boast that the interests of the patients come first in their economy, but this boast is hypocritical so long as they maintain a system the object of which is to make, for a small financial return, tickets, which are essential to admission, artificially scarce. Patients, in these days, have sufficient difficulty in finding a bed at all, without being forced to waste their strength first in a wearisome and degrading search for subscribers' tickets. Anyone with an ounce of judgment ought to refuse to subscribe or leave money to any hospital which maintains the discredited ticket system. It probably turns, as it is, many bequests away from the hospitals which still maintain it.

### A BIG STEP AT SHEFFIELD.

The boards and staffs of the four associated hospitals in Sheffield have confirmed the arrangements made with Mr. Bernard A. Firth to purchase the Norton Hall Estate of 120 acres, at the price of £25,000, for the development of the voluntary hospitals. On completion Mr. Firth will present the hall and its immediate grounds to the purchasers. A cordial vote of thanks has been passed to the donor, and the Sites Committee of the Sheffield Joint Hospitals Council, of which the chairman is Mr. Simpson, has received a hearty acknowledgment of its advisory services in the matter. Norton Hall, by the way, is a house of some history. Mr. Firth has lived there since 1902.

